# Supplementary material for: Simultaneous motor preparation and execution in a last-moment reach correction task
Source: Nat Commun. 2019 Jun 20;10:2718. doi: 10.1038/s41467-019-10772-2 (PMC6586876; doi:10.1038/s41467-019-10772-2)
Supplement: Supplementary file 1 — Supplementary Information [file 41467_2019_10772_MOESM1_ESM.pdf]

Supplementary Information

Simultaneous motor preparation and execution in a last-moment reach correction task

KC Ames, SI Ryu, KV Shenoy

Supplementary Figure 1

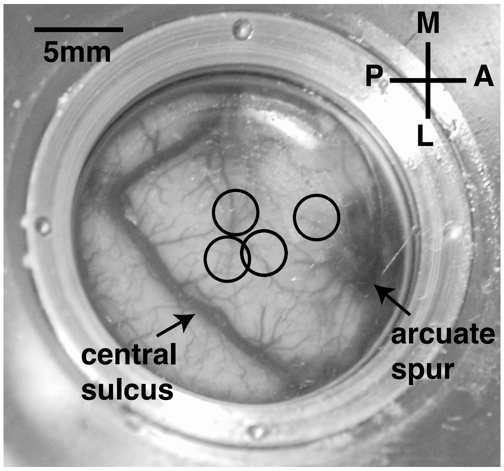

Recording locations, Monkey S. Burr hole locations for Monkey S superimposed on surface anatomy picture taken after subsequent craniotomy. For array implant locations for Monkey K see Ames et al, 2014.

Supplementary Figure 2

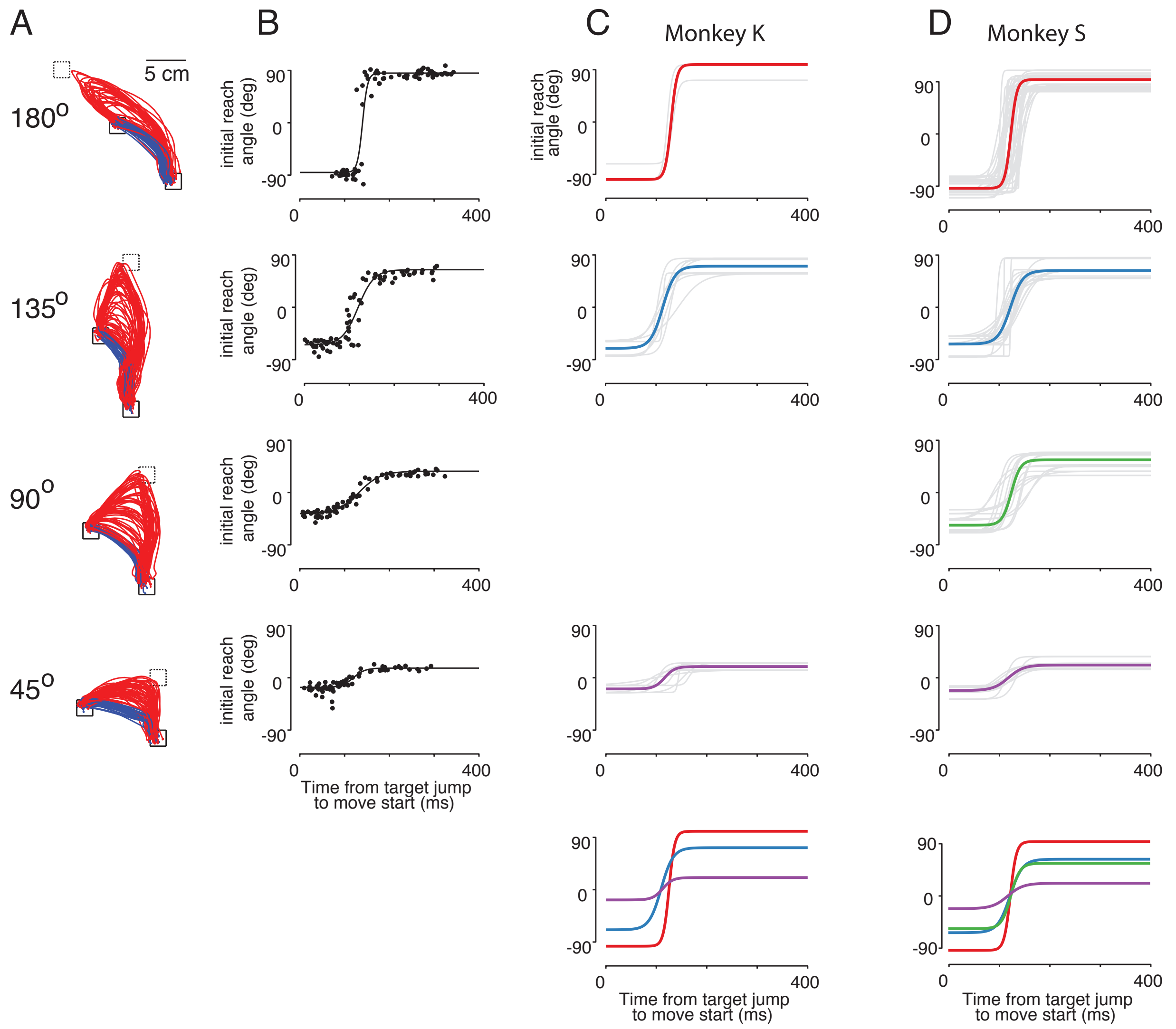

Initial reach angles after a target jump, non-normalized angles. (A) Reach paths following a target jump, for example conditions with a 180 degree, 135 degree, 90 degree, and 45 degree distance between targets. Red traces were initiated toward the initial target location and corrected online, blue traces were initiated toward the new target location. (B) Initial reach angle as a function of time from the target jump to movement onset, for the example conditions shown in A. Each dot shows one trial, lines show sigmoidal fit. (C-D) Sigmoidal fits for initial reach angles vs. time from target jump to movement onset, for all recorded conditions, for Monkey K (C) and Monkey S (D). Colored lines show average fits. Final column shows the overlap of average fits for each jump angle. Note that Monkey K did not perform 90-degree target jump conditions, so that entry is left blank.

Supplementary Figure 3

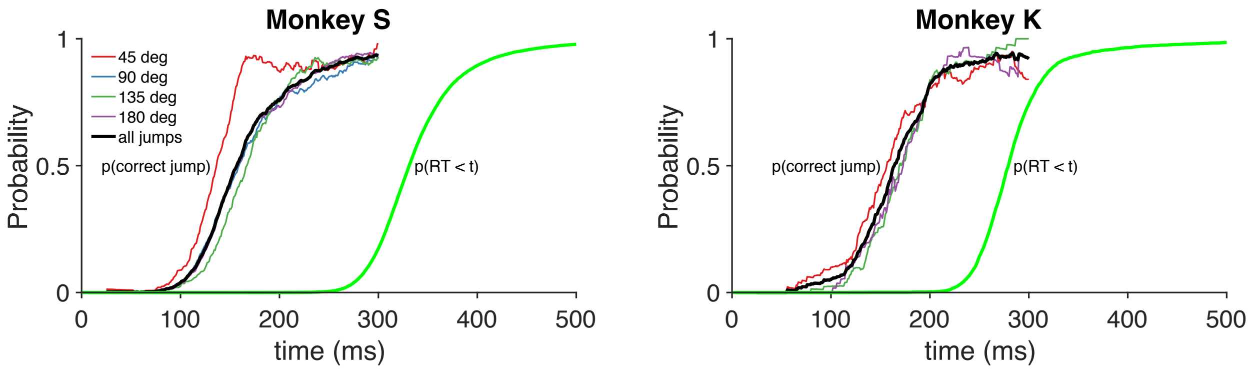

Time required to initiate a reach correctly after a target jump, compared to RT distribution for non-jump trials. Bright green line: Cumulative probability of moving after the go cue, calculated from all non-jump trials. Remaining colored lines: If movement was generated at a given time after the target jump, probability that that movement would be correctly initiated toward the new target. Probabilities calculated in 50-ms bins centered at each time point, across all trials with the given angle between jumps. Black line shows the probability calculated from all target jump trials, regardless of angle.

Supplementary Figure 4

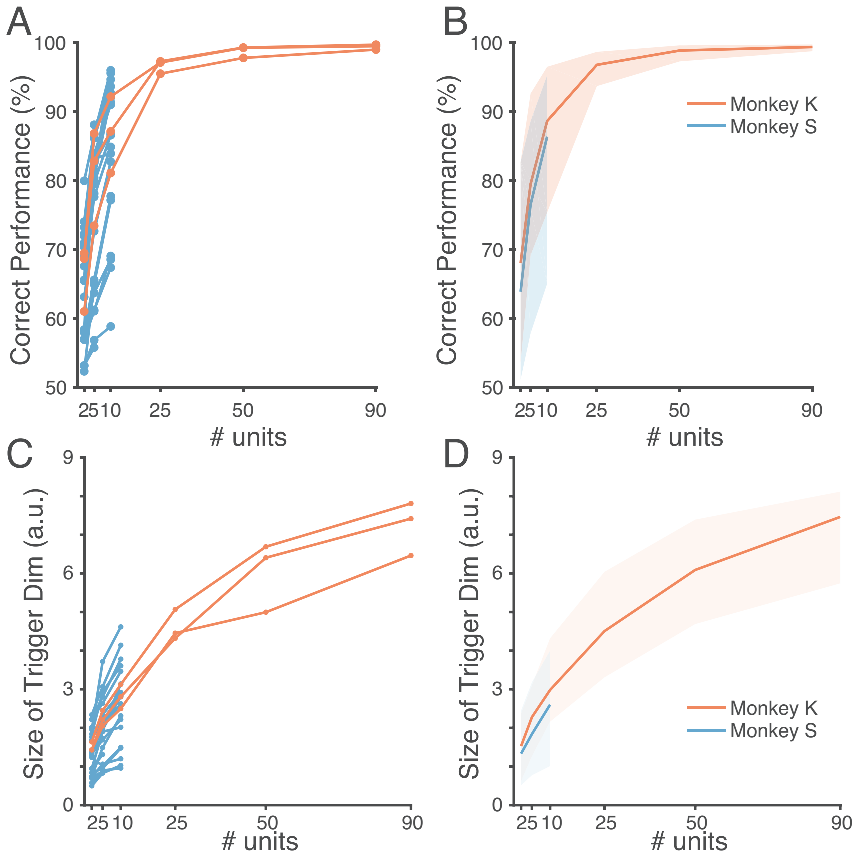

Trigger dimension properties versus number of units used. To determine whether performance differences between monkeys K and S trigger signal identification could be caused by fewer simultaneous units in Monkey S, we performed repeated sub-selections of units from our datasets and repeated our trigger-signal analysis. We first assessed how well neural data from held-out non-jump trials could be classified using a subset of 2,5,10,25, or 90 units (Monkey K), or a subset of 2,5, or 10 units for Monkey S. For each dataset and each subsample size, the subsampling was repeated 10 times. (A) Average performance for each dataset, across 10 resamplings for each size. Orange lines: Monkey K. Blue lines: Monkey S. (B) Median performance across all datasets and resamples. Shaded areas show 10th and 90th percentiles of the distribution. (C) For the same trigger signal identification as in A-B, the size of the trigger dimension versus the number of units. The size of the trigger signal was defined as the range of values achieved for the average trajectory in the trigger dimension from 500 ms before movement onset to 500 ms after movement onset (as displayed for the full dataset in Figure 5A-B). (D) Median performance across all datasets and resamples.

Supplementary Figure 5

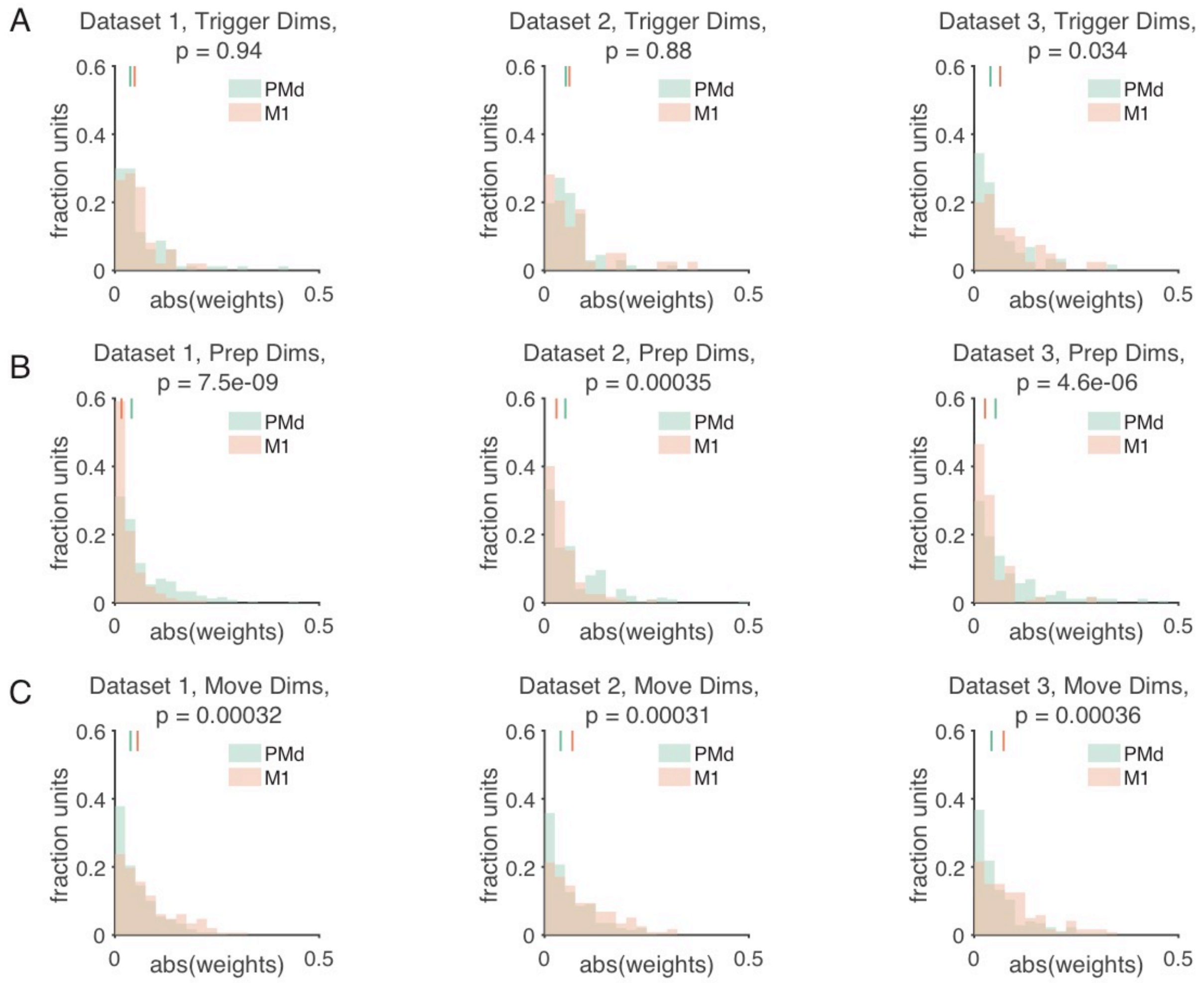

Distribution of weights across M1 and PMd units. In Monkey K, units were simultaneously recorded in M1 and PMd. We therefore examined the trigger dimension weighs for units from Monkey K's datasets to determine if one area preferentially contributes to the trigger,prep, and move dimensions. (A) Each panel shows the distribution of the absolute value of trigger dimension weights across units, for M1 (orange) and PMd (green). Lines indicate the medians of the distributions. P-values calculated using a two-sided Wilcoxon rank sum test. (B) As in A, for the preparatory dimensions. (C) As in A and B, for the movement dimensions.

Supplementary Figure 6

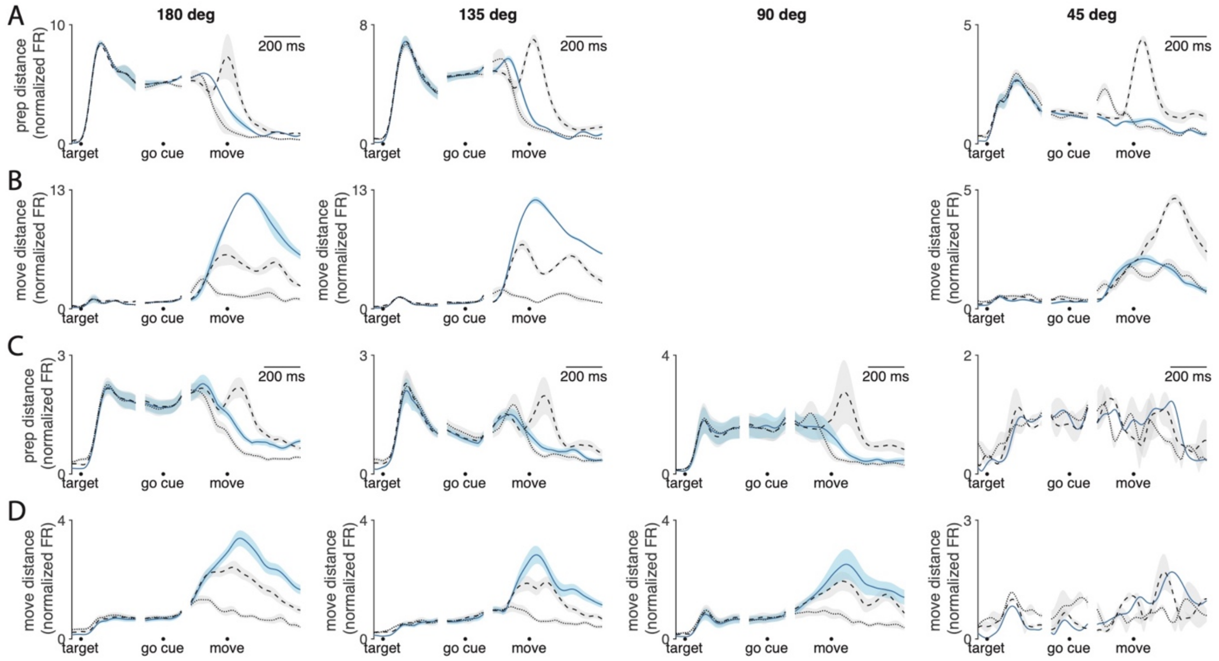

Target jump response in preparatory and movement dimensions, separated by reach angle. (A) For Monkey K, neural distance between the neural trajectory for reaches to the final target, for different reaching conditions. Blue: Distance between neural trajectories for non-jump reaches to the first and second target. Dotted line: Neural distance for correctly-initiated jump reaches. Dashed line: Neural distance for incorrectly-initiated jump reaches. All lines are mean +/- standard error across conditions. (B) As in A, for neural distance in movement-related dimensions. (C-D) As in A-B, for Monkey S.
