# Supplementary material for: Simultaneous motor preparation and execution in a last-moment reach correction task
Source: Nat Commun. 2019 Jun 20;10:2718. doi: 10.1038/s41467-019-10772-2 (PMC6586876; doi:10.1038/s41467-019-10772-2)
Supplement: Supplementary file 3 — Reporting Summary [file 41467_2019_10772_MOESM3_ESM.pdf]

nature research

Corresponding author(s): Katherine Cora Ames, coraames@gmail.com

Last updated by author(s): May 24, 2019

Reporting Summary

Nature Research wishes to improve the reproducibility of the work that we publish. This form provides structure for consistency and transparency in reporting. For further information on Nature Research policies, see Authors & Referees and the Editorial Policy Checklist.

Statistics

For all statistical analyses, confirm that the following items are present in the figure legend, table legend, main text, or Methods section.

n/a	Confirmed	
☐	☒	The exact sample size (n) for each experimental group/condition, given as a discrete number and unit of measurement
☐	☒	A statement on whether measurements were taken from distinct samples or whether the same sample was measured repeatedly
☐	☒	The statistical test(s) used AND whether they are one- or two-sided<br>*Only common tests should be described solely by name; describe more complex techniques in the Methods section.*
☐	☒	A description of all covariates tested
☐	☒	A description of any assumptions or corrections, such as tests of normality and adjustment for multiple comparisons
☐	☒	A full description of the statistical parameters including central tendency (e.g. means) or other basic estimates (e.g. regression coefficient) AND variation (e.g. standard deviation) or associated estimates of uncertainty (e.g. confidence intervals)
☐	☒	For null hypothesis testing, the test statistic (e.g. F, t, r) with confidence intervals, effect sizes, degrees of freedom and P value noted<br>*Give P values as exact values whenever suitable.*
☒	☐	For Bayesian analysis, information on the choice of priors and Markov chain Monte Carlo settings
☒	☐	For hierarchical and complex designs, identification of the appropriate level for tests and full reporting of outcomes
☐	☒	Estimates of effect sizes (e.g. Cohen's d, Pearson's r), indicating how they were calculated

Our web collection on statistics for biologists contains articles on many of the points above.

Software and code

Policy information about availability of computer code

Data collection	Data collection was performed using custom behavioral control and recording code written in Tempo and Matlab.
Data analysis	Custom code for data analysis was written in Matlab. Code for separating preparatory and movement dimensions was provided by Gamaledin Elsayed and John Cunningham, who developed the algorithm in Elsayed, Gamaleldin F., et al. "Reorganization between preparatory and movement population responses in motor cortex." Nature Communications 7 (2016): 13239.

For manuscripts utilizing custom algorithms or software that are central to the research but not yet described in published literature, software must be made available to editors/reviewers. We strongly encourage code deposition in a community repository (e.g. GitHub). See the Nature Research guidelines for submitting code & software for further information.

Data

Policy information about availability of data

All manuscripts must include a data availability statement. This statement should provide the following information, where applicable:

- Accession codes, unique identifiers, or web links for publicly available datasets
- A list of figures that have associated raw data
- A description of any restrictions on data availability

All relevant data can be made available by the authors on request.

Field-specific reporting

Please select the one below that is the best fit for your research. If you are not sure, read the appropriate sections before making your selection.

☒ Life sciences ☐ Behavioural & social sciences ☐ Ecological, evolutionary & environmental sciences

For a reference copy of the document with all sections, see nature.com/documents/nr-reporting-summary-flat.pdf

Life sciences study design

All studies must disclose on these points even when the disclosure is negative.

Sample size	We recorded data from two monkeys, which is standard for the field. From Monkey S, we recorded 288 units across 24 days. From Monkey K, we include 3 days' worth of data from chronic arrays; because units recorded are not guaranteed to be unique from day to day, each dataset is treated independently; Number of units per dataset in Monkey K ranged from 76 to 117 units. These sample sizes are comparable to prior studies of this area and topic.
Data exclusions	All single and multi-units which were well-isolated across a full recording session were included in our data. For Monkey S, population analyses were not performed on recording days with fewer than 10 simultaneously-recorded unit (5/24 days' datasets excluded)
Replication	All analyses were repeated separately for each of two monkeys.
Randomization	Trial order was randomized for both monkeys.
Blinding	Investigators were not blinded in this study.

Reporting for specific materials, systems and methods

We require information from authors about some types of materials, experimental systems and methods used in many studies. Here, indicate whether each material, system or method listed is relevant to your study. If you are not sure if a list item applies to your research, read the appropriate section before selecting a response.

Materials & experimental systems

n/a	Involved in the study	
☒	☐	Antibodies
☒	☐	Eukaryotic cell lines
☒	☐	Palaeontology
☐	☒	Animals and other organisms
☒	☐	Human research participants
☒	☐	Clinical data

Methods

n/a	Involved in the study	
☒	☐	ChIP-seq
☒	☐	Flow cytometry
☒	☐	MRI-based neuroimaging

Animals and other organisms

Policy information about studies involving animals; ARRIVE guidelines recommended for reporting animal research

Laboratory animals	2 adult male rhesus macaques (macaca mulatta)
Wild animals	n/a
Field-collected samples	n/a
Ethics oversight	All research protocols were approved by the Stanford Institutional Animal Care and Use Committee.

Note that full information on the approval of the study protocol must also be provided in the manuscript.
